# Children's understanding of demonstratives: an experimental study with German-speaking children between 5 and 7 years of age

**DOI:** 10.3389/fpsyg.2024.1403528

**Published:** 2024-08-14

**Authors:** Ramiro David Glauer, Elena Sixtus, Gregor Kachel, Jan Lonnemann, Frauke Hildebrandt

**Affiliations:** ^1^Social and Educational Sciences Department, University of Applied Sciences, Potsdam, Germany; ^2^Empirical Childhood Research, University of Potsdam, Potsdam, Germany; ^3^Institute of Educational Sciences, Leipzig University, Leipzig, Saxony, Germany

**Keywords:** demonstratives, language acquisition, deictic reference, pragmatic development, perspective taking

## Abstract

Demonstratives (“this”/“that”) express a speaker-relative distance contrast and need to be substituted for each other systematically: depending on their relative position, what one speaker refers to by saying “this” another speaker has to refer to by saying “that.” This substitution aspect of demonstratives poses additional difficulties for learning demonstratives, because it requires recognizing that two speakers have to refer to the same thing with different words, and might be one reason for the reportedly protracted acquisition of demonstratives. In an online study conducted in German, it was investigated whether children in the estimated upper age range of demonstrative acquisition (5 to 7 years) understand demonstratives' substitution aspect with familiar (“dies”/“das”) and novel (“schmi”/“schmu”) demonstratives, and whether they understand novel words (“schmi”/“schmu”) when used non-demonstratively as labels (*N* = 73; between-subject). Children's accuracy was compared with adult performance (*N* = 74). The study shows that children between 5 and 7 years of age perform less accurately than adults in all conditions. While adults' performance was highly accurate in all conditions (between 75% and 92% correct), children performed below chance in both demonstrative conditions and above chance in the labeling condition. This suggests that children do not understand demonstratives in the presented setup. More detailed analyses of children's response patterns indicate that they instead treat words as mutually exclusive labels in any condition.

## 1 Introduction

Demonstratives such as *this* and *that* are among the first words children use in their early language production, being often the first noncontent words uttered along with pointing gestures (Clark and Sengul, 1978; Kita, 2003; Diessel, 2006; but cf. González-Peña et al., 2020). Typically, demonstratives occur in pairs marking a distance contrast (“here”/“there”, “this”/“that”), and are thought to provide a conceptual frame of reference emerging prior to all other frames (Tanz, 1980). Linguistic research on spatial indexicals and psychological research on joint attention suggest that spatial indexicals constitute a universal class of expressions of fundamental significance for cognition and communication (Diessel, 2014; Diessel and Coventry, 2020). Demonstratives commonly occur together with an attention-directing gesture (e.g., pointing or gaze). They are language universal (Wierzbicka, 1996; Diessel, 2006; Levinson et al., 2018; Coventry et al., 2023). In exophoric use, they function as spatial deictic expressions to point to a location or an object relative to the deictic center, i.e., the speaker (Bühler, 1934; Coventry et al., 2008; Diessel, 2014). However, some researchers have proclaimed that demonstratives do not primarily function based on body-centered spatial frames of reference but serve to direct hearers' attention in various ways depending on physical, psychological, and referent-intrinsic factors of the situation (Levinson, 2003; Peeters and Özyürek, 2016; Peeters et al., 2021). Diessel and Coventry (2020) convincingly argued that the influence of various factors on a speaker's demonstrative choice is compatible with their universally encoding speaker-centered spatial relations.

Most languages worldwide use two or three terms to mark different distances from the speaker deictically and to refer to the same individual from different perceptual situations (Anderson and Keenan, 1985; Coventry et al., 2023). In German, the language the study was conducted in, there used to be a two-way distinction employing the proximal “dieser/diese/dies(es)”, corresponding to “this” or “this one” in English, and distal “jener/jene/jenes,” corresponding to “that” or “that one”.^1^ However, the distal term (“jener/jene/jenes”) got out of use in everyday exophoric usage. It is now common to mark the distance contrast by adding locative adverbs (“hier”, “da,” and “dort”) to the previously proximal demonstrative or to a definite article (“der/die/das”), effectively allowing a three-way distinction.^2^ But the contrast between a medial “der/die/das da” and a distal “der/die/das dort” (both roughly corresponding to “that one there”) appears to be less pronounced than in other three-way languages (Coventry et al., 2023). It is unclear whether the former proximal demonstrative (“dieser/diese/dies(es),” corresponding to “this” or “this one”) and the definite articles (“der/die/das,” corresponding to “the”) can be used contrastively without adding a locative adverb. While some researchers argue that “dieser/diese/dies(es)” might have lost its contrastive meaning because the contrast “jener/jene/jenes” is no longer used (Himmelmann, 1997; Diessel, 1999, 2006, 2013), there is no empirical evidence that “dieser/diese/dies(es)” is not used contrastively, for instance, contrasting the definite articles “der/die/das.” In effect, “dieser/diese/dies(es)” can be more naturally combined with the proximal locative adverb “hier,” while “der/die/das,” can be combined naturally with any locative. This suggests that “dieser/diese/dies(es)” retain some of their proximal meaning, especially if used in salient contrast to “der/die/das.” Thus, German may be in a process of diachronic change, i.e., grammaticalization, developing a new proximal-distal contrast pair with “dieser/diese/dies(es)” being used as the proximal form and “der/die/das” as the distal form.

Unlike other spatial expressions, demonstratives are very old, probably older than all other functional words (Diessel, 2014). They are the primary source of functional morpheme development and do not themselves derive from other word roots (Diessel, 1999, 2006; Diessel and Breunesse, 2020).

Despite their linguistic peculiarity and evident centrality to human communication, few studies investigated how children learn demonstratives. Extant studies suggest that adult-like uses of demonstratives are the result of protracted development (González-Peña et al., 2020), with various aspects of their meaning being learned subsequently (De Villiers and De Villiers, 1974; Webb and Abrahamson, 1976).

### 1.1 Experimental work on the acquisition of demonstratives

Empirical studies on the development of children's comprehension and production of the terms “this/that” are relatively sparse. Results from studies with English-speaking children suggest that this development extends over several years. De Villiers and De Villiers (1974) examined English-speaking children ranging in age from 2 to 4 years. Comprehension and production of “this” and “that” were tested in a hide-and-seek game where the experimenter sat beside or opposite the child. Results revealed that children as young as 3 years of age showed high performance rates (“this”: 80%, “that”: 91%) in the comprehension task that required translation from the experimenter's perspective to their own perspective. Performance in the production task differed as a function of the experimenter's sitting position; for example, 3-year-old children performed better when the experimenter sat next to them (“this”: 75%, “that”: 80%) than when the experimenter sat opposite them (“this”: 80%, “that”: 50%).

Webb and Abrahamson (1976) investigated the acquisition of the terms “this”/“that” in English-speaking children of ages 4 and 7 years, also in a comprehension and a production task. Children‘s performance in the comprehension task was better for the word “that” (“this”: 66%, “that”: 73%), it was better when experimenter and child had the same perspective (“this”: 83%, “that”: 68%) than when they faced each other (“this”: 48%, “that”: 78%), and it was better for the 7-year-old children (“this”: 73%, “that”: 78%) compared to the 4-year-old children (“this”: 58%, “that”: 67%).

Clark and Sengul (1978) presented English-speaking children aged 2 to 4 years with a game in which they had to decide which of two toys to move, testing their comprehension of demonstratives. The experimenter sat beside the child or on the opposite side of the table, facing the child. Children's performance increased with age (e.g., 3-year-olds: “this”: 56%, “that”: 51%; 4-year-olds: “this”: 75%, “that”: 76%), and children were more likely to answer correctly to the word “this” with the experimenter next to them (“this”: 83%, “that”: 42%), but were more frequently correct on the word “that' with the experimenter facing them (“this”: 51%, “that”: 85%). Only 5 of 36 children appeared to have a complete understanding of the terms “this” and “that.”

In a recent study by González-Peña et al. (2022), the production of the terms “this one/that one” was investigated in English-speaking children ages 7 and 11. In two experiments, children were asked to tell a puppet, supposedly understanding only the words “this one” and “that one,” which of two identical-looking dinosaurs had “jumped” from the side of the table to a position along a wooden bar extending from the child. The dinosaurs were distinguished by differently colored stickers, and before the experiments, children could choose one of the dinosaurs which they could keep afterwards. In the first experiment, the 11-year-old children used the term “this” more often for positions that were closer to them and the term “that” more often for positions that were further away from them. This was not the case for the 7-year-old children. In addition, there was a trend across both age groups to use the term “this” more often for the chosen dinosaur, irrespective of its distance from the child. In a second experiment, the stimulus value was increased by combining the dinosaur figure with a token of economic value. This resulted in a stronger effect of “ownership” compared to the first experiment (the term “this” was used significantly more often for the chosen dinosaur). Moreover, not only the 11-year-old children but also the 7-year-old children used the term “this” more frequently for positions closer to them and the term “that” more frequently for positions farther away from them. According to González-Peña et al. (2022), these results suggest that distinctions in demonstrative production emerge around the age of 7, assuming sampling differences as the reason for the variation between experiments.

In sum, the results of studies exploring the development of comprehension and production of the terms “this/that” in English-speaking children are inconclusive. English-speaking children may begin to distinguish between these terms in comprehension and production as early as age 3 (De Villiers and De Villiers, 1974), although other findings suggest that this does not occur until age 7 (Webb and Abrahamson, 1976; González-Peña et al., 2020).

Findings from studies with children speaking languages other than English also do not allow to draw clear conclusions regarding the development of comprehension and production of the terms analogous to “this/that.” Comprehension of such terms was found to be above chance in 5-year-old Mandarin-speaking children, even when the terms were uttered by a speaker with a different perspective (Chu and Minai, 2018). Regarding production, Turkish-speaking children have been reported to acquire the basic distinction between specific terms for objects that are close and far from themselves at age 4 (Küntay and Özyürek, 2006). In contrast, this competence has been reported in 6- to 8-year-old but not in 3- to 5-year-old Spanish-speaking children (Shin and Morford, 2020).

### 1.2 Theoretical considerations

Based on a detailed review of extant findings of adults' use of demonstratives, Peeters et al. (2021) argued that speakers' choice of demonstrative form is not primarily influenced by speaker-centric distance to the referent. Whether a proximal or a distal demonstrative is used may, for instance, be influenced by hearers' or the dyad's distance to the referent, by its visibility, assumed relevance, possession, or other non-spatial factors. Moreover, there might be non-contrastive uses of demonstratives. The variability of speakers' choice of demonstrative form is taken to suggest that speaker-centric distance is not central to demonstratives' meaning. However, while the actual choice of a demonstrative and its interpretation appear to depend on various semantic and pragmatic factors, Coventry et al. (2023) demonstrated that the speaker-centric distance contrast is a language-universal meaning aspect of demonstratives. And while they found hearer-centric uses of demonstratives in their study, these were only found for some languages and remained comparably rare. It is safe to conclude that a referent's distance to the speaker is a central aspect of demonstratives in exophoric usage even if the choice of demonstrative form may also be influenced by other factors in actual conversations such that what counts as near may, for instance, include the shared space between conversation partners or a larger region around the speaker or the speaker-hearer dyad (Bühler, 1934; Diessel, 2014).

From the fact that demonstratives mark a relative distance contrast to the speaker it follows that speakers have to use different words to refer to the same thing from different perspectives and may have to refer to different things using the same word from different perspectives. What one speaker refers to by saying “this” or “here,” another speaker, standing some distance away, must refer to by saying “that” or “there”—equally in the case of just one speaker who changes her position. If used in a distance-contrasting way, demonstratives need to be *substituted* for each other to refer to the same thing from different positions.

Consider what is generally involved in learning new words and what is peculiar about learning demonstratives. While content words can be learned by associating words with featurally discriminable aspects of the environment, demonstratives require sensitivity to a higher-level similarity. Because anything can be this or that, the referents of “this” and “that” have nothing in common except for their relative distance to whoever is speaking. Moreover, note that language learners need not know that two uses of “this” and “that” refer to the same thing. For one, as argued by Hildebrandt and Glauer (2023) and Hildebrandt et al. (2023), children might lack the required ability to individuate objects before being able to use demonstratives adequately, and only through learning demonstratives an abstract frame of reference for object individuation is acquired. For another, even if children do individuate objects early on Van de Walle et al. (2000), Stavans et al. (2019), and Xu and Carey (1996), they might have difficulties relating two uses of “this” and “that”. Arguably, this should be quite common in actual conversations where several objects can be at the right relative distances from speakers. Moreover, children (as well as adults) have a bias toward using one word per object when encountering novel words with unknown meaning (Halberda, 2003, 2006; Markman et al., 2003; mutual exclusivity bias, Merriman et al., 1989).

Correspondingly, identifying the right speaker-relative distances is not sufficient for bringing together uses of “this” and “that” referring to the same thing from different positions. In addition to the relative distance contrast, learners must relate uses of demonstratives adequately. Such related uses of demonstratives differ from corresponding uses of content words. For instance, one speaker might ask someone: “Could you pass me that apple, please?” To which the other person might respond: “This one?” And the answer would be: “Yes, that one.” Here, we have an affirmative behavior following subsequent uses of different demonstratives. A similar pattern of uses of different words can be viable for content words, such as when one speaker uses a superordinate category word or a description (e.g., “long-furred, purring predator”) in response to a request involving some base category word (e.g., “cat”). But in the case of demonstratives, it is mandatory if used contrastively either to use the same or another demonstrative, depending on relative positions. Demonstratives form a closed class of words which have to be *substituted* in characteristic ways. That is, to say the same thing, speakers at different positions must replace one demonstrative by another.

The importance of demonstrative substitution can easily be overlooked, because it appears to be a direct consequence of demonstratives' relative-distance contrast. Once speakers have focused on a shared object of attention, demonstrative substitution follows from each speakers' choosing demonstratives according to the focused-on object's distance. However, as argued by Hildebrandt and Glauer (2023), assuming that speakers' focus of attention can be conceptualized as *the same object* at the outset of learning demonstratives underestimates the complexity of proper object individuation which requires sensitivity to objects' individuation criteria. Object individuation requires unequivocally distinguishing all particular objects. Because there might be two objects that are totally alike in all their discearnible features, such as two industrially produced screws of the same make, object individuation requires more than perfect similarity. Ultimately, what distinguishes each object is its spatial position relative to other objects at the same time. Thus, for ordinary material objects, the individuation criteria are spatiotemporal, and the ability to individuate objects requires “anchoring” them spatially and temporally in an adult like way, i.e., irrespective of sorting them by similarity. By learning a substitutional system of demonstratives, an intersubjectively shared, abstract frame of reference is acquired that goes beyond the distinction of what is reachable or non-reachable for someone by combining several speakers' perspectives within an intersubjective coordinate system. Such a coordinate system can be used to localize, and thereby individuate, proper objects (Tugendhat, 1976, 2016; Hildebrandt and Glauer, 2023; Hildebrandt et al., 2023).

In sum, spatial indexicals comprise the following decisive semantic features: they are context-sensitive in that their referents change from situation to situation. They mark a distance contrast relative to the speaker. And they must be substituted for each other to refer to the selfsame object from different positions. To our knowledge, the substitution aspect has not yet been investigated. Test conditions involving one or more speakers talking about the same thing from different positions would allow probing children's understanding of the substitution aspect of spatial indexicals' meaning.

### 1.3 This study

The study was conducted in German employing the terms “dies” and “das” without adding the locative adverb. The speaker could be referring to one of two alike-looking objects at different distances on a table, making a two-way contrast salient—even if the contrastive meaning of the German demonstratives, when used individually, is not as strong as in other languages, for instance, of “this” and “that” in English. “Dies” and “das” were the natural choice because we were aiming to focus on the objects referred to, not their location.

To test not only the substitution knowledge of children incorporated in the concrete terms “dies” and “das” but also to determine whether children can employ a systematic rule-based substitution that goes beyond the context of familiar words, we chose to integrate a pseudo-word condition. Pseudo-words such as “Schmi,” i.e., phonologically viable forms that are not in the lexicon of a given language, are used extensively in linguistic and psycholinguistic experiments. Children who encounter pseudo-words cannot rely on their existing vocabulary or experience with these words. By comparing children's performance with pseudo-words and actual words, we aim to conclude whether children possess a flexible understanding of the semantic substitution structures of indexicals independent of specific words.

As target objects, we have used two identical-looking balls placed symmetrically on a table with no further distinguishing features in the background to ensure that participants make responses based on the objects' distances to the speaker, not based on an object's salience or preferences for an object's or its surrounding features. The study employs the one-speaker version of demonstrative substitution. That is, in the whole experiment, one speaker referred to the target objects from different positions. The study was restricted to one-speaker substitution because participants might assume that two speakers use the same words with slightly different meanings, presenting the additional difficulty of having to decide whether they employ the same or alternative meanings.

As a control, we have devised a condition in which children are not forced to include the speaker's spatial perspective and which does not require the ability to substitute expressions. This condition resembles a labeling or naming condition in which a word such as “cat” has to be associated with an object kind. However, because no external distinguishing features are apparent in the objects to be labeled, the task is more complex than ordinary labeling. It requires considering objects' position and resembles tasks involving relational properties (e.g., grandma's favorite ball) or dispositional properties (e.g., magnetism). Such tasks are referred to here as *complex labeling tasks*.

Studies show that the development of comprehension and production of the terms “this/that” occurs primarily between 3 and 7 years of age (see above). Since substitution is a demanding semantic feature, we want to investigate whether children in the upper range of the age window are capable of demonstrative comprehension, including substitution. In addition, we have included adults to estimate proficient performance in the presented tasks.

Finally, if not employing demonstratives' meaning rules, children might follow different response strategies. Children might select responses randomly. However, there is evidence in the literature suggesting that children prefer selecting objects closer to the speaker (Clark and Sengul, 1978). Moreover, it was argued that children have a mutual exclusivity bias when learning new content words (Merriman et al., 1989; Halberda, 2003, 2006; Markman et al., 2003). This bias might likewise be effective when learning demonstratives.

### 1.4 Hypotheses

Consistent with existing studies of demonstrative comprehension and following from the complexity of demonstratives' substitution aspect, we assumed that children (5–7) do not fully master the usage rules of ordinary nor pseudo-word demonstratives. Moreover, we assume that children of that age have remaining difficulties with complex labeling tasks. Thus, we hypothesize that children perform significantly less accurately than adults in all conditions (H1).

Children in the upper acquisition age range should partly understand demonstratives' meaning. They might, nonetheless, lack a flexible understanding of the semantic substitution structures of demonstratives. Understanding pseudo-word demonstratives, independent of already learned words, would thus be more difficult for children than understanding demonstratives. Therefore, we assume that children perform significantly more accurately in the demonstrative condition than in the pseudo-word demonstrative condition (H2).

Moreover, the complex labeling condition does not require a substitution rule. Therefore, we hypothesize that children perform significantly more accurately in the complex labeling condition than in both other conditions (H3).

Concerning children's response strategies, three hypotheses were formulated *post-hoc* and tested in an exploratory analysis. First, children might select responses randomly (R1, random choice). Second, children might tend to select the ball closer to the speaker (R2, proximity bias). Third, for the second request in a trial, children might tend to select the ball not selected in their first response (R3, mutual exclusivity bias).

## 2 Methods

### 2.1 Participants

A total of 73 children and 74 adults were tested in a 2 × 3 between-subjects design (see Table 1). The total sample size was based on standard practices in the field. With this sample size, G*Power sensitivity analyses indicated that *t*-tests testing our three main hypotheses would be able to detect large effect sizes of Cohen's d ≥ 0.87 (H1: three one-tailed *t*-tests with Bonferroni-corrected alpha-level of 0.05/3, power of 80%, group sizes of 24 and 25 participants each), Cohen's d ≥ 0.72 (H2: one one-tailed *t*-test with an alpha level of 0.05, power of 80%, and group sizes of 24 and 25 participants), and Cohen's d ≥ 0.82 (H3: two one-tailed *t*-tests with Bonferroni-corrected alpha-level of 0.05/2, power of 80%, and group sizes of 24 and 25 participants). Children were recruited online, in Potsdam, a medium-sized Central European city, or in a test center in Blossin/Heidesee (Brandenburg, Germany), being visited by kindergarten groups from Berlin, and the State of Brandenburg. Children in Potsdam were contacted via a database of participants for child development studies to which their parents had voluntarily signed up. Appointments were made based on parents' and children's availability. The socio-economic status of families was not recorded. Still, the test center is visited by children with diverse backgrounds, generally representing the ethnic and socio-economic range of a suburban-to-rural region in Central Europe. All studies described below were reviewed and approved by an internal ethics committee at the University of Potsdam. Adult participants were recruited online. Data collection took place from March to December 2021. In addition, 11 children (6 female) were tested but not submitted to the final sample for not fitting into the planned age range.

**Table 1:** Demographics - age categories by condition.

**Age**	**Condition**	**N**	**female**	**M age**	**Min age**	**Max age**	**SD age**
Adult	Complex labeling	25	19	34.80	20	62	11.66
Adult	Demonstrative	24	14	32.12	21	70	12.33
Adult	Pseudo-word demonstrative	25	19	33.72	19	61	12.23
Child	Complex labeling	24	13	5.50	5	7	0.59
Child	Demonstrative	25	9	5.56	5	7	0.65
Child	Pseudo-word demonstrative	24	14	5.38	5	6	0.49

The table shows per condition and age group the number of participants (N), the number of female participants (female), the mean age (M age), the minimum and maximum age (Min age and Max age), and the standard deviation of participants' age (SD age).

### 2.2 Procedure and setup

Following the rules at the University of Applied Sciences Potsdam during the SARS-CoV-2 pandemic, the study was conceptualized as an online experiment. The study was advertized through social media channels and email lists. Parents could either follow a link and let their children participate in the experiment whenever convenient (*N* = 6), or they were asked to make an appointment for a video call during which an experimenter assisted in the experiment (*N* = 5). Due to difficulties in the online acquisition of participants, the study was conducted with mobile devices in a test center in Blossin/Heidesee (Brandenburg, Germany) when regulations allowed face-to-face contact with children (*N* = 60). In the online version, the experiment began with a written introduction for parents, explaining how the experiment was conducted and expressing that children could break off participation at any time. In the face-to-face version, experimenters explained the experiment and the possibility of leaving at any time to the participating children. Parents or experimenters helped children operate the device on which experiments ran. All adults were tested online.

Three conditions were tested between-subject. In all conditions, video recordings of an experimenter demonstrated how a pair of words is used to refer to two visually identical balls lying on the table before her. The conditions were distinguished by which pair of words was used and by these words' meaning rule. In the first condition, the demonstrative condition, “dies” (German for “this”) and “das” (German for “that”) were used in their ordinary usage (see above). In the second condition, the pseudo-word demonstrative condition, the pseudo-words “schmi” and “schmu” were used with the meaning rule of demonstratives. In the third condition, the complex labeling condition, the same pseudo-words were used with a simpler meaning rule requiring only the temporary association of the word to a ball on one side of the table. The experimenter then expressed a preference for one of the balls, and during a still image, children were asked to click the preferred ball. The children observed the scene side-on (see Table 1). Three variables with two values each were counterbalanced for the trials, giving eight trials per condition. The trials varied in whether the experimenter started from the left or right side, whether she introduced “schmi” or “schmu” (“this” or “that”) first, and whether she asked for schmi or schmu (this or that) first. In the pseudo-word demonstrative condition, schmi was always the proximal object to match the phonetic properties of “this” vs. “that”.

### 2.3 Stimulus material

**Figure 1:**
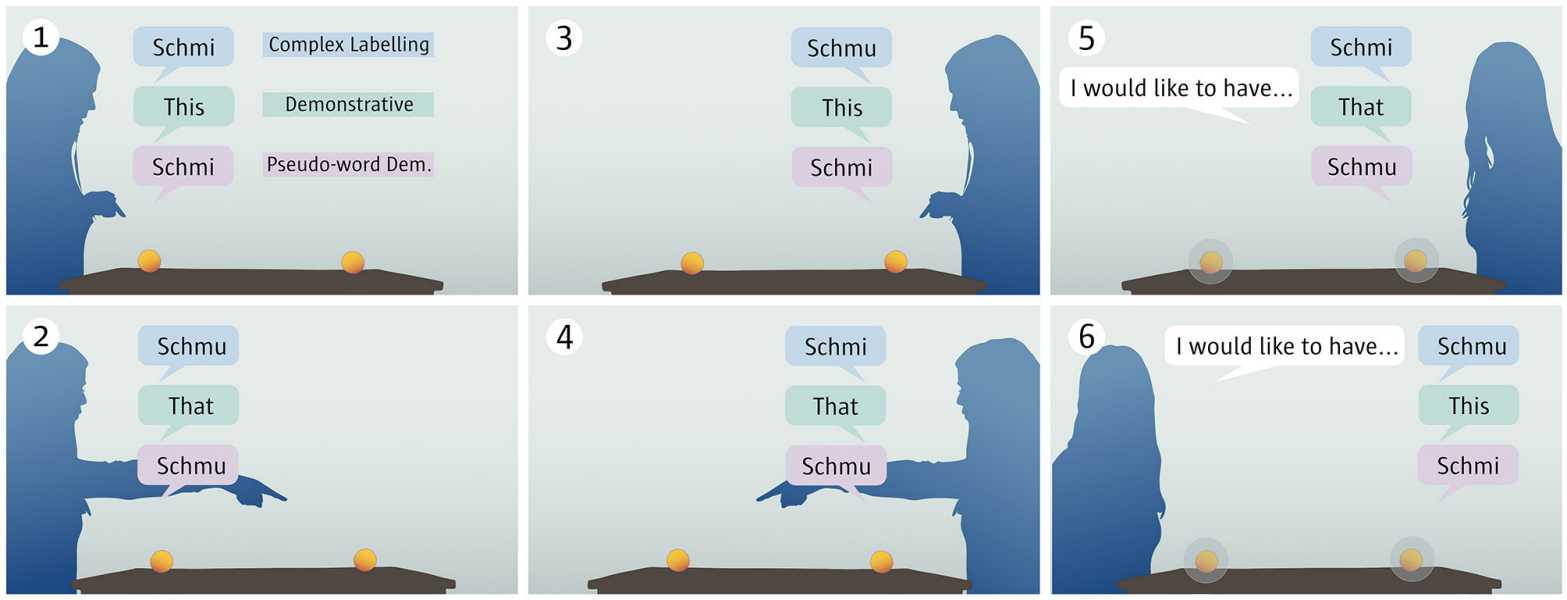
Schematic presentation of experimental setup in the three conditions. In the experiment, the sides (left/right) from which the experimenter started, the first demonstrated word (this/that, schmi/schmu), and the first word used in the request were counterbalanced between trials.

In each trial, participants were presented with a series of brief video sequences in which an experimenter demonstrated the meaning of a pair of words (“schmi”/“schmu” or “this”/“that”) pointing at one of two identical-looking balls. The balls were placed symmetrically on a table, and the experimenter first demonstrated what she meant by each word from one side of the table and then from the other. The experimenter then changed sides again and said she would like to have schmi/schmu (this/that). Participants were then asked to click the ball the experimenter wanted to have (see Figure 1).

### 2.4 Coding

Understanding the substitution aspect of demonstratives requires understanding that the same object must be referred to using different words from different perspectives. Thus, an understanding of the substitution aspect of demonstratives is indicated by an ability to select the same ball in subsequent requests employing other words from different standpoints. Correspondingly, an understanding of “schmi” and “schmu” in the complex labeling condition requires being able to select different balls for subsequent requests employing other words. Thus, in all conditions, only when the target ball was successfully selected for both requests in a trial was it coded as a success. Participants' proportion of correct trials was used as an estimate of participants' proficiency in the task.

For response strategy R1 (random choice), the mean of correct responses for individual requests and for trials was calculated for each participant.

For response strategy R2, a proximity bias index was calculated for each child by subtracting the number of distal choices from the number of proximal choices and dividing the result by the number of requests. Because responses were forced-choice, the index ranges from –1 to 1. Positive values indicate a bias toward selecting the proximal ball. Negative values indicate a preference for the distal ball.

For response strategy R3, an index for the mutual exclusivity bias was calculated for each child by subtracting the number of trials in which children chose the same ball twice from the number of trials in which they chose different balls and dividing the result by the number of trials. Thus, the index ranges from –1 to 1. Positive values indicate a bias toward treating the target words as mutually exclusive labels. Negative values indicate a tendency to apply both words to the same object.

### 2.5 Analyses

Because the proportion of correct trials per participant was not normally distributed, hypotheses were tested using directed Wilcoxon rank sum tests instead of *t*-tests. Response strategy R1 was tested using two-tailed Wilcoxon rank sum tests against chance. If children respond randomly to each request, they should choose each ball with an equal probability of 50% in each request. Trials should correspondingly be answered correctly with a probability of 25% because each trial consists of two requests which would be answered independently. Response strategies R2 and R3 were tested using two-tailed Wilcoxon rank sum tests against a neutral index of 0. All analyses were run in R (R Core Team, 2022) using the stats package for analyses and ggplot2 (Wickham, 2016) and ggsci (Xiao, 2023) for visualization. Data and manuscript were prepared using the tidyverse and papaja packages (Wickham et al., 2019) and are available online.

## 3 Results

### 3.1 Overview

**Figure 2:**
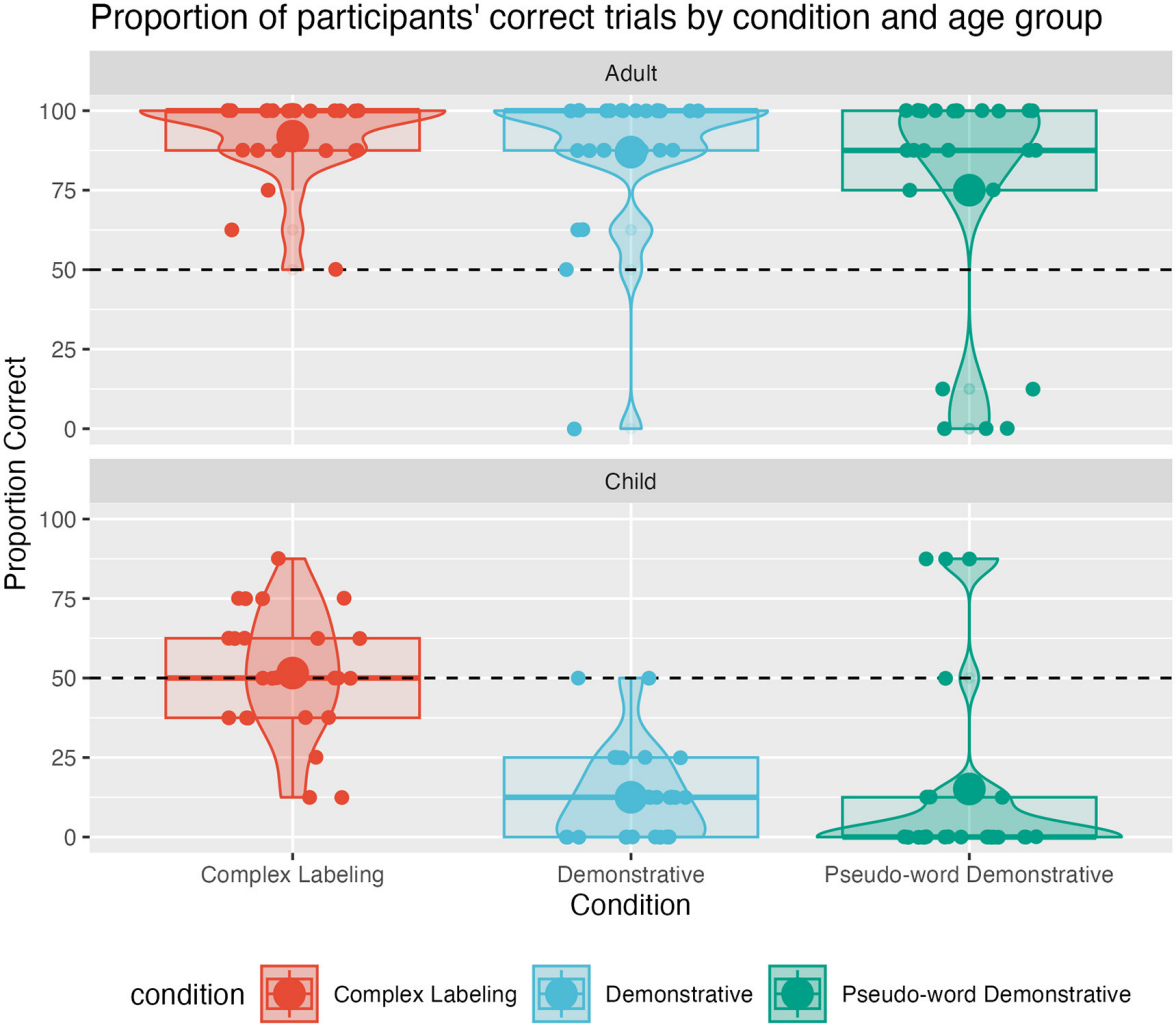
Distribution of participants' percentage of correct trials per condition and age group.

Adults gave 92% correct answers in the complex labeling condition, 86.98% correct answers in the demonstrative condition, and 75% correct answers in the pseudo-word demonstrative condition. This is interpreted as proficient performance in the presented task. Children performed less successfully in all conditions (complex labeling: 51.56%; demonstrative: 12.50%; pseudo-word demonstrative: 15.10%) (see Table 2, Figure 2).

**Table 2:** Overview of performance in test trials.

**Age**	**Condition**	**N**	**trials/N**	**M**	**SD**
Adult	Complex labeling	25	8.00	92.00	12.95
Adult	Demonstrative	24	8.00	86.98	23.16
Adult	Pseudo-word demonstrative	25	8.00	75.00	36.80
Child	Complex labeling	24	8.00	51.56	19.61
Child	Demonstrative	25	8.00	12.50	14.88
Child	Pseudo-word demonstrative	24	8.00	15.10	29.94

The table provides the number of participants (N) for all experimental groups, as well as the percentage (M) and standard deviation (SD) of correct choices. Data for each child were averaged across trials.

### 3.2 Hypotheses testing

#### 3.2.1 H1: Children perform significantly less accurately than adults in all conditions

Children perform significantly less accurately than adults in the complex labeling condition (*W* = 573.00, *p* < 0.001, Δ*Mdn* = 0.38, 95% CI [0.37, ∞]), in the pseudo-word demonstrative condition (*W* = 522.00, *p* < 0.001, Δ*Mdn* = 0.87, 95% CI [0.75, ∞]), as well as in the demonstrative condition (*W* = 579.50, *p* < 0.001, Δ*Mdn* = 0.87, 95% CI [0.75, ∞]). H1 could be confirmed.

#### 3.2.2 H2: Children perform significantly more accurately in the demonstrative (dies/das) condition than in the pseudo-word demonstrative (schmi/schmu) condition

Results revealed no significant difference between children's performance in the demonstrative and pseudo-word demonstrative conditions (*W*= 355.00, *p*= 0.114).

#### 3.2.3 H3: Children perform significantly more accurately in the complex labeling condition than in both other conditions

Directed Wilcoxon rank sum tests showed that responses were more accurate in the complex labeling than in the pseudo-word demonstrative condition (*W* = 490.50, *p* < 0.001, Δ*Mdn* = 0.50, 95% CI [0.37, ∞]) as well as in the complex labeling than in the demonstrative condition (*W* = 558.50, *p* < 0.001, Δ*Mdn* = 0.38, 95% CI [0.37, ∞]). H3 could be confirmed.

### 3.3 Response strategies

#### 3.3.1 R1: Random choice

Table 3 shows the probabilities of responding correctly for each request and for trials. Two-sided Wilcoxon signed rank tests on children's responses to the first and second requests (Bonferroni-corrected α-level of 0.05/6) suggest that children pick balls randomly in the demonstrative and pseudo-word demonstrative conditions because the probability of correct responses is not significantly different from a chance level of 50% for all requests. In the complex labeling condition, children answer correctly significantly above chance to first requests. Moreover, children's trial performance significantly differs from chance in the complex labeling and demonstrative conditions (Bonferroni-corrected α-level of 0.05/3). It is above chance for complex labeling (M = 45, *p* = 0.0848) and below chance in the demonstrative (M = 55.21, *p* = 0.309) condition. This suggests that children did not select balls randomly response-by-response in all conditions. In the complex labeling condition, children seem to understand the meaning of the novel words partly. In the demonstrative condition, the second response appears to be influenced by the first, even if first responses result from guessing, leading to below-chance performance.

**Table 3:** Average probability of children's correct answers for first requests, second requests, and trials.

**Condition**	**Response**	**N**	**M**	**SD**	**p**
Complex labeling	correct_first	24	63.54	19.82	0.00541*
Complex labeling	correct_second	24	57.81	17.99	0.0583
Demonstrative	correct_first	25	45.00	13.50	0.0848
Demonstrative	correct_second	25	57.50	16.14	0.0334
Pseudo-word demonstrative	correct_first	24	49.48	21.33	0.937
Pseudo-word demonstrative	correct_second	24	55.21	23.58	0.309
Complex labeling	correct_trial	24	51.56	19.61	< 0.001**
Demonstrative	correct_trial	25	12.50	14.88	0.00301**
Pseudo-word demonstrative	correct_trial	24	15.10	29.94	0.0438

The table provides the number of participants (N) for all experimental groups, as well as the percentage (M) and standard deviation (SD) of correct choices. Two-sided Wilcoxon rank sum tests tested group-level performance against a chance-level of 50% for individual requets and of 25% for trials. The asterisks indicate significant effects (Bonferroni-corrected; trials: *p < 0.0167, **p < 0.0033, ***p < 0.00033; responses: *p < 0.0083, **p < 0.0017, ***p < 0.00017).

#### 3.3.2 R2: Proximity bias

Children were not overall biased toward choosing the ball closer to the speaker (complex labeling: M = −0.01, SD = 0.44, W = 124.50, *p* = 0.961; demonstrative condition (M = 0.26, SD = 0.53, W = 205.50, *p* = 0.0401; pseudo-word demonstrative: M = 0.05, SD = 0.50, W = 136.50, *p* = 0.472; two-sided Wilcoxon rank sum tests with Bonferroni-corrected α-level of 0.05/3 against a proximity bias of 0).

**Figure 3:**
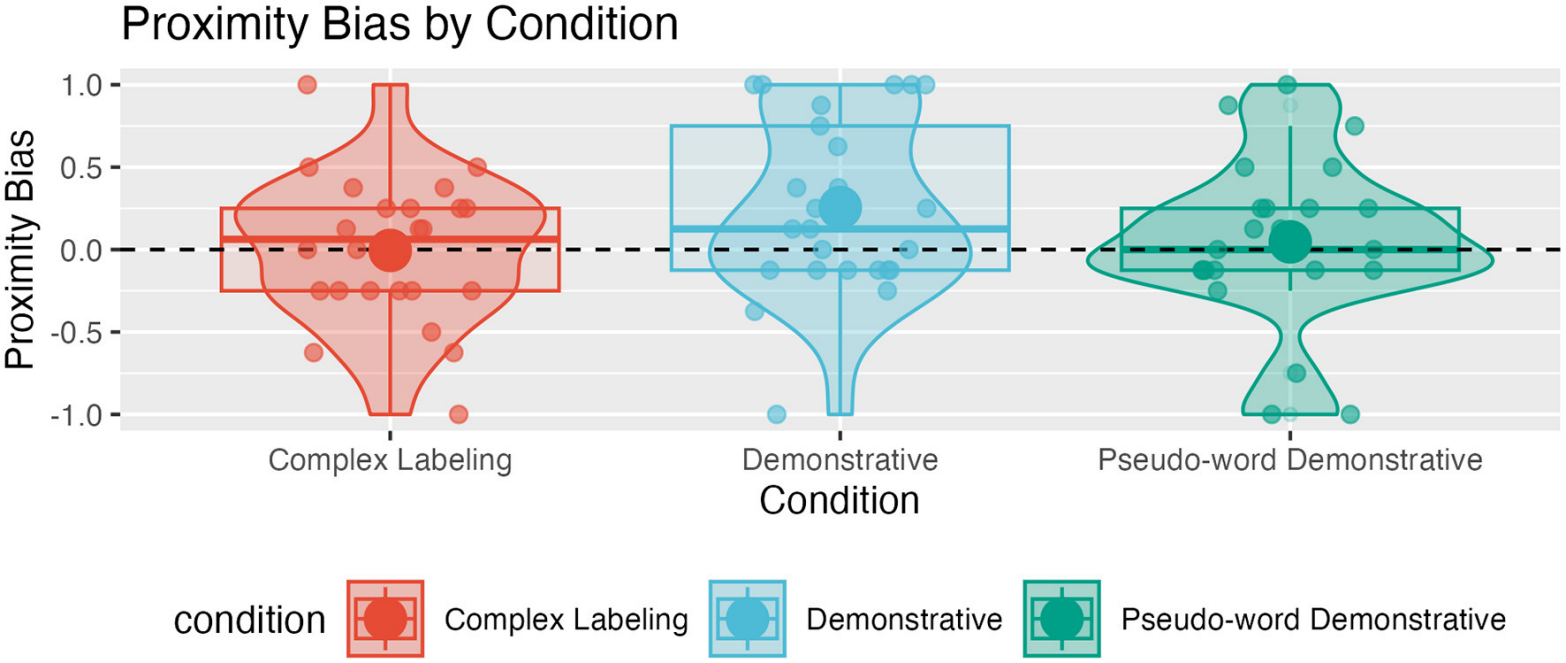
Distribution of participants' proximity bias index; positive values indicate that participants selected the speaker-proximal ball more often than the distal ball, negative values indicate that participants more often select the distal ball.

The distribution of index values suggests that individual children follow different strategies when selecting responses. Most children do not have a dominant preference for proximal or distal balls. Nevertheless, in each condition, some children always choose either the proximal or distal ball (see Figure 3).

#### 3.3.3 R3: Mutual exclusivity bias

In the current setup, the mutual exclusivity bias is useful when interpreting “schmi” and “schmu” in the complex labeling condition. In this condition, each word corresponds to one ball at a certain position—irrespective of where the speaker stands. However, associating each word with one of the balls in this way is incompatible with the meaning of demonstratives.

**Figure 4:**
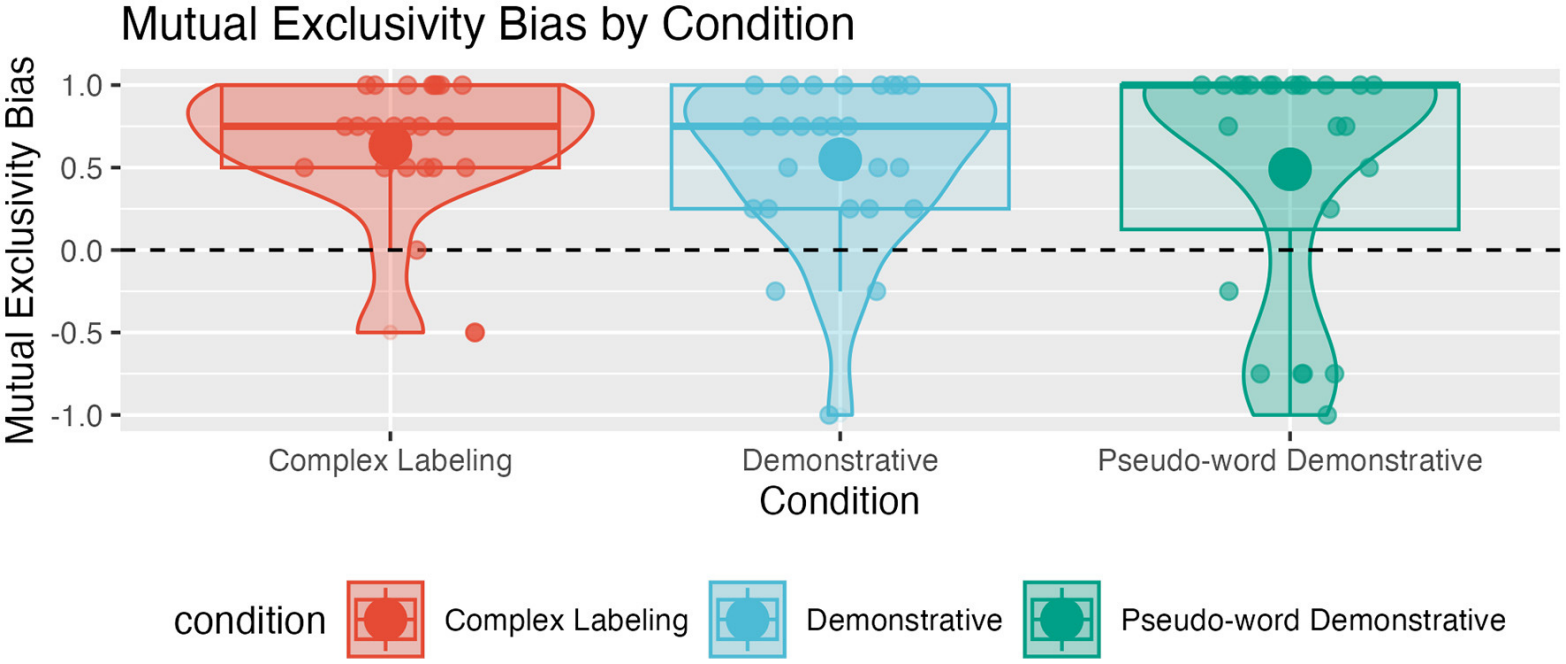
Distribution of participants' mutual exclusivity bias index; positive values indicate that participants switch selected balls between requests within one trial more often than not, negative values indicate that participants more often select the same ball twice; the size of dots represents participants' proportion of correct answers.

In most cases, children tend to select different balls within trials. The probability of selecting both balls in a trial significantly differs from chance in the complex labeling and demonstrative conditions, but not in the pseudo-word demonstrative condition (complex labeling: M = 0.64, SD = 0.43, W = 267, *p* ≤ 0.0001; demonstrative: M = 0.55, SD = 0.50, W = 296, *p* = 0.000302; pseudo-word demonstrative: M = 0.49, SD = 0.74, W = 253, *p* = 0.00263; two-sided Wilcoxon rank sum tests with Bonferroni-corrected α-level of 0.05/3 against a mutual exclusivity bias of 0; see Figure 4).

The index values distribution suggests that most children tend to treat words as mutually exclusive labels. This tendency is strongest in the complex labeling condition, which is consistent with the meaning of labels. It is weakest and not significant in the pseudo-word demonstrative condition. However, many children exhibit an extreme mutual exclusivity bias in this condition (Median and Mode = 1), and some show a reversed tendency to select the same ball twice within a trial. The latter is consistent with the demonstrative meaning of “schmi” and “schmu” in this condition. Together with the distribution of correct answers showing that some children have high success rates in this condition (see Figure 2), this suggests that some children have understood the demonstrative meaning of “schmi” and “schmu” while others adher to treating the words as mutually exclusive labels.

## 4 Discussion

### 4.1 Response correctness rates

The results show that 5–7-year-old children perform less accurately than adults in all conditions. In particular, they have difficulties understanding spatial demonstratives (12.50% correct trials) and pseudo-word spatial demonstratives (15.10% correct trials). This corresponds to the expectations derived from extant findings on the acquisition of demonstratives (De Villiers and De Villiers, 1974; Webb and Abrahamson, 1976; Clark and Sengul, 1978; González-Peña et al., 2020) and the theoretically derived complexity of demonstratives' substitution aspect (Tugendhat, 1976, 2016; Hildebrandt and Glauer, 2023; Hildebrandt et al., 2023).

The assumption that pseudo-word demonstratives are even more challenging to understand than ordinary demonstratives could not be confirmed for the tested age range. Using pseudo-words does not appear to make recognizing the substitution rule more difficult. As argued, this would be expected if children in that age range had already partially understood demonstratives, allowing them to answer some requests correctly that involve familiar demonstratives. Because correctness rates are similarly low in both conditions, we interpret these findings as showing that children aged 5 to 7 years not only lack a flexible understanding of the semantic substitution features of demonstratives but do not understand demonstrative substitution at all under tested conditions. The substitution rule of demonstratives appears to pose a major obstacle for learning demonstratives.

As hypothesized, children perform significantly more accurately in the complex labeling condition than in both other conditions. Nonetheless, children answer correctly significantly less often than adults. We interpret these results as follows. In the case of complex labeling, children need not apply a substitution rule. The tendency to use one word per object when confronted with novel words (mutual exclusivity bias) suffices to perform correctly in the complex labeling condition. However, this condition differs from simple labeling—such as when children learn words for different kinds of animals: the labeled objects have the same superficial properties and differ only in their position in space. Moreover, no other differences were presented, like dispositional properties (magnetism, ability to emit sounds), ownership (my ball, Marie's ball), or specific histories that could have been used to mark each ball differentially. The lack of (easily observable or unobservable) distinguishing features complicates correct labeling. Therefore, children still find it more difficult than adults to understand requests in the complex labeling condition.

Overall, 5- to 7-year-old German-speaking children do not appear to understand the meaning rule of demonstratives, nor do they apply a labeling rule to the full extent.

### 4.2 Children's response strategies

The first analysis of children's responses suggests that children predominantly selected their first response randomly in the demonstrative and pseudo-word demonstrative conditions. In the complex labeling condition, first responses and trials were correct above chance, suggesting that children understood the presented labels at least in some cases and did not respond randomly. The second analysis shows that children did not tend to select the proximal ball. The third analysis revealed that children tended not to select the same ball twice within a trial in the complex labeling and demonstrative conditions. In the complex labeling condition, children selected the correct ball above chance in their first response and the other in their second response. This is compatible with the intended meaning of “schmi” and “schmu” as labels in this condition. While children appeared to follow the correct labeling rule in the complex labeling condition, their performance remained below the level of adults. As argued above, we take this to stem from the complexity involved in understanding that two distinct labels are used for two identical-looking objects.

In the demonstrative condition, children chose their first response randomly and then adhered to selecting the other ball in response to the second request as well—which is not compatible with the contrastive meaning of demonstratives. This suggests that children did not interpret “dies” (“this”) and “das” (“that”) as demonstratives in these conditions and treated them as labels instead. In the pseudo-word demonstrative condition, children overall appeared to answer randomly. However, the distribution of individual biases shows that many children had a strong tendency to select both balls while some children had a clear tendency to select the same ball twice.

Overall, the pattern of responses suggests that children had a strong tendency to follow a labeling rule when interpreting the meaning of novel (pseudo-)words, or even familiar words, as indicated by their mutual exclusivity bias. When words are not used as labels—as in the demonstrative and pseudo-word demonstrative conditions—children resort to guessing.

### 4.3 Children's understanding of demonstratives

The current findings indicate that 5- to 7-year-old German-speaking children were below adult levels of accuracy when interpreting demonstratives, pseudo-word demonstratives, and complex labels in the present experimental setup. This shows that children have difficulties understanding demonstratives' substitution aspect and is in line with extant findings suggesting that the acquisition of demonstratives is the result of a protracted learning process that extends into the early elementary school years (Webb and Abrahamson, 1976; Chu and Minai, 2018; González-Peña et al., 2020; Shin and Morford, 2020).

Moreover, analyses of response strategies suggest that children responded randomly in their first response when interpreting ordinary demonstratives or pseudo-word demonstratives and that subsequent responses followed a rule adequate for learning labels such as classifying expressions or names. When confronted with two words and two objects, it is functional to assume that the two words are mutually exclusive, each labeling one thing. However, such a labeling rule is incompatible with the meaning of demonstratives. Understanding demonstratives consists in knowing that “this” is to be systematically substituted for “that” when referring to the same thing from different speaker positions (Tugendhat, 1976, 2016). This indicates that German-speaking children between 5 and 7 years of age do not fully understand demonstratives under tested conditions.

Because mutual exclusivity is incompatible with the meaning of demonstratives, it is striking that children tend to interpret words as mutually exclusive labels even when used with a demonstrative meaning. In our interpretation, this indicates an underlying tendency to understand words as labels for things in one's environment. Such labels can be learned by associating words with (kinds of) objects. However, in the case of demonstratives, the substitution rule gives the only stable association, and it holds between words: demonstratives can refer to anything but must be systematically substituted for each other depending on the speakers' positions (Hildebrandt and Glauer, 2023).

### 4.4 Limitations, open questions, and further research

Due to the restrictions during the COVID-19 pandemic, this study was planned to be conducted online. Therefore, the experimental stimuli had to be recorded and presented on screen. Videotaping stimuli, as opposed to live performance by experimenters, also had the advantage of controlling the stimuli fully. However, recorded scenes might be less engaging than interactions with an experimenter conducting the study. In particular, video-recorded verbal interactions might be less engaging than proper interactions with an experimenter, especially in the case of demonstratives, which usually serve to establish shared attention to a common referent in a shared environment, making it more difficult for children who have not yet reached a fully flexible understanding of demonstratives. Therefore, we recommend that future research on demonstratives employ live interactions with experimenters in the same room.

In the current study, “dies” and “das” were used as distance-contrastive demonstratives without adding the locative adverbs “hier” and “da,” although including “hier” and “da” might have been the natural choice given the widespread conviction that demonstratives lack a contrastive meaning in German. However, as argued above, German might be in the process of grammaticalization, developing a contrastive meaning of “dies” and “das”. Adults' tendency to interpret “dies” and “das” contrastively under tested conditions suggests that “dies” and “das” are naturally interpreted in a distance-contrastive way. Irrespective of the present research questions, it would be worth investigating whether “dies” and “das” are used contrastively in German.

Moreover, the current study suggests that German-speaking children between 5 and 7 years of age do not understand demonstratives. Participating children did not only have difficulties with the semantic feature of substitution but did not interpret demonstratives correctly, even in their first response. This suggests they did not understand demonstratives at all (see Section Overview). As a result, the acquisition age of the substitution aspect of demonstratives' meaning could not be determined within this study. To determine how demonstratives are learned, the age range should be extended in a way corresponding to possible facilitations of the task associated with in-person testing. Moreover, different semantic features should be investigated separately (several items for context sensitivity, distance contrast, speaker-relativity, and substitution, see Section Theoretical considerations). Despite the above reasoning for using only one speaker (see Section This study), for completeness, the substitution aspect should also be tested with several speakers referring to the same thing from different positions.

### 4.5 Conclusion

The study suggests that German-speaking children between 5 and 7 years of age do not understand the substitution aspect of demonstratives. This means they do not understand that different words must be used to refer to the same thing from different positions, depending on speakers' relative distance to the referents. Instead, analyses of children's response patterns suggest that they tend to interpret words as mutually exclusive labels, even when used as demonstratives. These results complement findings showing that children acquire a comprehensive understanding of demonstratives rather late, focusing on the substitution aspect that is particularly crucial for demonstratives' meaning. That demonstratives are learned rather late is plausible at the outset, because the rules for using demonstratives are much more complex than for content words. While content words can be learned by associating words with featurally discriminable aspects of the environment, demonstratives require sensitivity to a more abstract similarity. Anything can be this or that. The referents of “this” and “that” have nothing in common except for their relative distance to whoever is speaking. The meaning of demonstratives is grounded in an initial understanding of distance as reachability together with a sensitivity to when one demonstrative (“this” or “that”) must be replaced by another (“that” or “this”).

If the thesis is correct that children lack the ability to individuate objects before being able to use demonstratives adequately, and that only through learning demonstratives an abstract frame of reference is acquired, allowing proper object individuation (Hildebrandt and Glauer, 2023; Hildebrandt et al., 2023), the current findings suggest that children between 5 and 7 years have not developed an adult-like spatial reference system that allows them to develop identity criteria for objects in the full sense.

The current findings are relevant for a more comprehensive understanding of the acquisition of demonstratives, representing a special class of function words. The complexity of demonstratives' usage rules and children's tendency to use different words for different objects partly explains children's protracted learning of demonstratives, at the same time being among the first words used by L1-acquiring children and being used in an adult-like way only after several years. Further research will have to investigate the developmental acquisition order of all semantic aspects of demonstratives separately with children from a more extensive age range and in a more realistic setting to determine when and how demonstratives are learned by L1-acquiring children.

## Data availability statement

The datasets presented in this study can be found in online repositories. The names of the repository/repositories and accession number(s) can be found at: https://github.com/rahmiro/glauer_etal_2024_understanding-demonstratives.

## Ethics statement

The studies involving humans were approved by the Ethics Committee of the University of Potsdam. The studies were conducted in accordance with the local legislation and institutional requirements. Written informed consent for participation in this study was provided by the participants or their legal guardians/next of kin.

## Author contributions

RG: Conceptualization, Formal analysis, Investigation, Project administration, Visualization, Writing – original draft, Writing – review & editing. ES: Data curation, Formal analysis, Investigation, Methodology, Visualization, Writing – original draft. GK: Data curation, Formal analysis, Investigation, Methodology, Visualization, Writing – original draft. JL: Investigation, Methodology, Supervision, Writing – original draft, Writing – review & editing. FH: Conceptualization, Methodology, Project administration, Resources, Writing – original draft, Writing – review & editing.

## Footnotes

^1^In German, demonstratives and articles correspond to grammatical gender, presented here in the order (m/f/n).

^2^“hier” is proximal, corresponding to “here” in English, “dort” is distal, corresponding to “there”. “da” is used to mark a medial distance and is commonly translated as “there” as well.
